# Phenotypic Divergence of P Proteins of Australian Bat Lyssavirus Lineages Circulating in Microbats and Flying Foxes

**DOI:** 10.3390/v13050831

**Published:** 2021-05-04

**Authors:** Celine Deffrasnes, Meng-Xiao Luo, Linda Wiltzer-Bach, Cassandra T. David, Kim G. Lieu, Lin-Fa Wang, David A. Jans, Glenn A. Marsh, Gregory W. Moseley

**Affiliations:** 1 Department of Microbiology, Biomedicine Discovery Institute, Monash University, 19 Innovation Walk (Bldg 76), Melbourne, VIC 3800, Australia; Celine.Deffrasnes@monash.edu (C.D.); cassandra.david@monash.edu (C.T.D.); 2 Department of Biochemistry and Molecular Biology, Bio21 Molecular Science and Biotechnology Institute, 30 Flemington Road, The University of Melbourne, Melbourne, VIC 3010, Australia; luo.m@wehi.edu.au (M.-X.L.); kim.g.lieu@gmail.com (K.G.L.); 3 Department of Biochemistry and Molecular Biology, Biomedicine Discovery Institute, Monash University, 19 Innovation Walk (Bldg 77), Melbourne, VIC 3800, Australia; linda.wiltzer@icloud.com (L.W.-B.); David.Jans@monash.edu (D.A.J.); 4 Programme in Emerging Infectious Disease, Duke-NUS Medical School, 8 College Road, Singapore 169857, Singapore; linfa.wang@duke-nus.edu.sg; 5 SingHealth Duke-NUS Global Health Institute, 8 College Road, Singapore 169857, Singapore; 6 Australian Centre for Disease Preparedness, CSIRO Health and Biosecurity, 5 Portarlington Road, East Geelong, VIC 3220, Australia; Glenn.Marsh@csiro.au

**Keywords:** Australian bat lyssavirus, lyssavirus, rabies virus, immune evasion, nuclear trafficking, interferon, STAT1, bats, virus reservoirs, adaptation

## Abstract

Bats are reservoirs of many pathogenic viruses, including the lyssaviruses rabies virus (RABV) and Australian bat lyssavirus (ABLV). Lyssavirus strains are closely associated with particular host reservoir species, with evidence of specific adaptation. Associated phenotypic changes remain poorly understood but are likely to involve phosphoprotein (P protein), a key mediator of the intracellular virus–host interface. Here, we examine the phenotype of P protein of ABLV, which circulates as two defined lineages associated with frugivorous and insectivorous bats, providing the opportunity to compare proteins of viruses adapted to divergent bat species. We report that key functions of P protein in the antagonism of interferon/signal transducers and activators of transcription 1 (STAT1) signaling and the capacity of P protein to undergo nuclear trafficking differ between lineages. Molecular mapping indicates that these differences are functionally distinct and appear to involve modulatory effects on regulatory regions or structural impact rather than changes to defined interaction sequences. This results in partial but significant phenotypic divergence, consistent with “fine-tuning” to host biology, and with potentially distinct properties in the virus–host interface between bat families that represent key zoonotic reservoirs.

## 1. Introduction

Bats are reservoirs for viruses, including henipaviruses, several coronaviruses, and most, if not all, members of the genus *Lyssavirus*, and carry many viruses that are pathogenic in other animals without developing disease, indicative of a specialized relationship [1,2]. Cross-species transmission (CST) of viruses from bats poses significant threats to human health through the emergence of novel diseases, particularly where spillover results in further transmission or sustained maintenance in new hosts. CST from zoonotic reservoirs without further transmission can also present a major burden; for example, humans are dead-end hosts for lyssaviruses that cause rabies, an invariably lethal acute meningoencephalitis, but rabies results in c. 59,000 human deaths each year [1,3,4]. This is largely due to the transmission of rabies virus (RABV) from dogs, although infections also include RABV and other lyssaviruses transmitted from bats and other mammals [1,2].

Lyssaviruses comprise at least 17 species (International Committee on Taxonomy of Viruses (ICTV)) and numerous lineages/strains. The species are classified within three or more phylogroups that have distinct serologic reactivity [1,4]. Cross-reactivity from RABV vaccines is evident within phylogroup 1 (that includes RABV and Australian bat lyssavirus (ABLV)) but not more distantly related lyssaviruses [1,4]. RABV/lyssaviruses are thought to cause disease in most, if not all, mammals, but epidemiological and experimental data indicate that species or strains/lineages are adapted to particular host species, with CST typically resulting in dead-end infections [1,4]. Notably, lyssaviruses can cause lethal disease in reservoir hosts, so adaptation does not appear to derive from major changes in the virus–host interface causing loss of pathogenesis but rather from a balance of the virus–host dynamic enabling persistence in the population [3,4]. As lyssaviruses share a relatively broad tissue tropism and largely indistinguishable pathogenesis, adaptation probably involves subtle phenotypic changes, including at the molecular virus–host interface to “fine-tune” strains to their host [4]. The nature of these changes remains poorly understood.

Among lyssaviruses, RABV uniquely exists in independent cycles in multiple volant and nonvolant mammalian species, where the association of each host with a specific strain and absence of circulation of the same strain in multiple hosts is consistent with historical CST events and adaptation [1,3,4]. Notably, RABV variants circulate in terrestrial mammals and, in the New World (i.e., the Americas) only, in diverse bat species [4]. In contrast, while most non-RABV lyssaviruses are maintained in bats, they have not been detected in New World bats and are maintained in a single species or highly restricted species range [4]. RABV is considered the main cause of human rabies, but CST of other lyssaviruses occurs, including in countries considered rabies-free such as Australia, where ABLV has infected humans and horses [1,5,6,7]. Misdiagnosis, underreporting, and a lack of discriminatory diagnostics suggest estimates of deaths due to RABV are conservative and likely to include more non-RABV lyssavirus infections than currently assumed. This highlights risks to public health in the absence of pan-lyssavirus protection by current vaccines [4].

**Figure 1:**
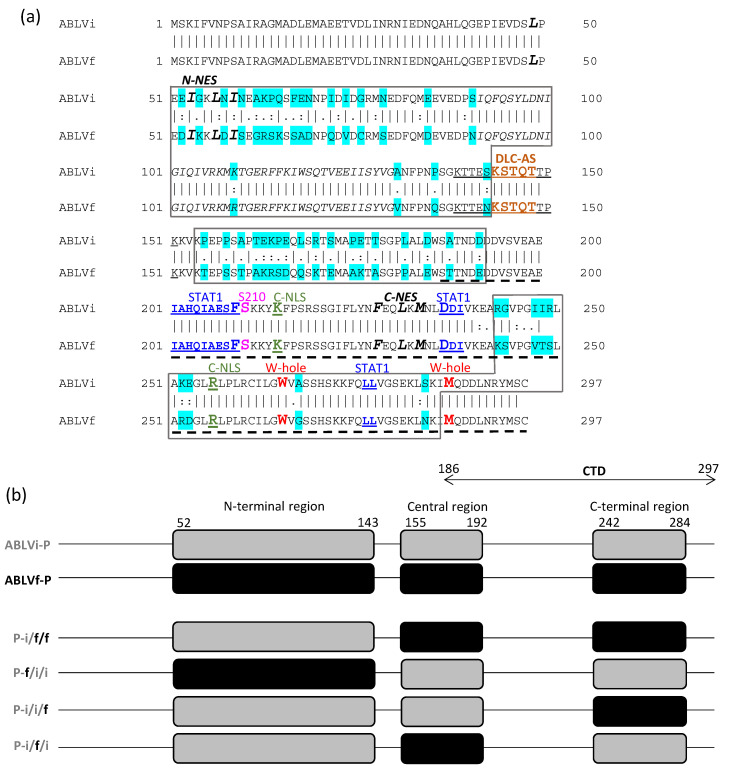
Sequence alignment of Australian bat lyssavirus (ABLV) phosphoproteins (P proteins) and schematic of P proteins and chimeras generated. (**a**) Alignment of sequences of ABLVi and ABLVf P proteins used (ABLVi and ABLVf indicate ABLV is from insectivorous or frugivorous bats, respectively); turquoise shading indicates substitutions between the proteins, with boxes indicating clusters of substitutions in the N-terminal (residues 52–143), Central (residues 155–192), and C-terminal (residues 242–284) regions. Key residues and sequences are indicated, including sequences important to nuclear trafficking: N-terminal nuclear export sequence (N-NES) motif (residues 49–58, hydrophobic residues of motif in the large bold italicized font); dynein light chain-association sequence (DLC-AS) motif (within residues 139–151, underlined; KSTQT motif is in the bold tan font); C-terminal nuclear localization sequence (C-NLS); key residues K214 and R260 in the large bold green font and underlined); S210 protein kinase C (PKC) site (large bold pink font); C-terminal NES (C-NES; residues F227, L230, M232 in the large bold italicized font); sequences implicated in signal transducers and activators of transcription 1 (STAT1) binding: STAT1-binding surfaces (201–209, 235–237, 276–277, bold/underlined blue font, with residues F209 and D235 in the large font); W-hole residues W265 and M287 (large bold red font). (**b**) Schematic representation of ABLVf, ABLVi, and chimeric P proteins generated. The globular C-terminal domain (CTD) is indicated (dotted line in (**a**), CTD in (**b**)).

Lyssavirus P protein is a multifunctional protein with critical roles in replication as an essential polymerase cofactor and at the intracellular virus–host interface, where its best-characterized roles are in evasion of antiviral signaling by interferon (IFN) cytokines [8,9]. Following infection, cells release type-I IFNs (IFNα/β), which induce phosphorylation of signal transducers and activators of transcription 1 (STAT1) at a conserved tyrosine (Y701), generating pY-STAT1. pY-STAT1 translocates to the nucleus and, in association with pY-STAT2 and IFN-regulatory factor 9 (IRF9), activates the transcription of IFN-stimulated genes (ISGs) [10,11,12,13]. The globular C-terminal domain (CTD) of P protein (Figure 1a) binds pY-STAT1 to inhibit signaling [14,15,16,17]. P protein also undergoes nuclear trafficking via several nuclear localization (NLS) and export (NES) sequences (see Figure 1a; [9,18,19,20,21]). These were identified in RABV P protein, but critical residues and trafficking are broadly conserved among lyssaviruses [22]. As lyssavirus replication is cytoplasmic, nuclear trafficking is thought to enable host modulation, including STAT1 antagonism [8,9].

The mechanisms of P protein immune evasion, and established or proposed roles of nuclear trafficking, are reviewed in detail elsewhere [8,9] and summarized briefly here. In full-length P protein (the most abundant isoform in infected cells [23]), a dominant N-terminal NES (N-NES) effects strongly cytoplasmic localization at steady state (due to prominent nuclear export activity), while a NLS (C-NLS) in the CTD enables nucleocytoplasmic shuttling [19,20]. A C-terminal NES (C-NES) and protein kinase C (PKC, S210) phosphorylation site, and a dynein light chain association sequence (DLC-AS) also regulate trafficking (Figure 1a). Additional sequences important in smaller isoforms are not active in full-length P protein [24,25]. The N-NES enables IFN antagonism by effecting the nuclear export/cytoplasmic arrest of P protein-associated pY-STAT1 such that the STAT1-binding site and N-NES of P protein are critical to immune evasion and are pathogenesis factors [15,22,26]. Nuclear import has also been implicated, as the binding of P protein to STAT1 inhibits DNA interaction, an intranuclear event [21]. However, the precise significance of P protein nuclear import in STAT1 antagonism and pathogenesis remains unresolved.

P protein shows the greatest sequence diversity among lyssavirus proteins [27] such that phenotypic changes in P protein–host interactions might be expected to arise between viral species. Indeed, while nuclear trafficking and STAT1 antagonism are broadly conserved, consistent with important roles in different hosts, quantitative differences exist between certain lyssavirus species [16,22], including increased nuclear import activity of P protein of Mokola virus (phylogroup 2) compared with phylogroup 1 viruses and reduced STAT1-antagonist function of P protein of Duvenhage virus (DUVV) compared with RABV. Whether such divergence exists between closely related lineages, including lineages associated with different bat species, is not known. ABLV provides a unique model among non-RABV lyssaviruses to assess this, as it cocirculates as two lineages (ABLVi and ABLVf) that appear to have evolved from a single progenitor and are associated with hosts differing at the family level (insectivorous and frugivorous bats, respectively) [1,3,5,28]. Here, we report that nuclear trafficking and STAT1 antagonist functions are maintained between ABLVi and ABLVf P proteins but with significant and independent quantitative differences. Key interaction sequences are not altered such that differences appear to involve distinct regions resulting in subtle changes, consistent with fine-tuning to the host. The identification of differences between closely related species is significant to lyssavirus biology, as well as indicating possible differences in the virus–host relationship between bat species.

## 2. Materials and Methods

### 2.1. DNA Constructs, Cell Culture, and Transfections

cDNA encoding the P protein was amplified by PCR using isolates of ABLV held within the Commonwealth Scientific and Industrial Research Organization (CSIRO) Australian Centre for Disease Preparedness. ABLVi P protein was amplified using an isolate from yellow-bellied sheath tail bat (GenBank Accession Number AF081020) and ABLVf P protein using an isolate from pteropid bat (GenBank Accession Number AF006497). The coding sequences were inserted into pEGFP-C1 in-frame C-terminal to green fluorescent protein GFP to produce GFP-fused P proteins, as previously described [22]. This results in the fusion of GFP at the N-terminal end of P protein, distant from the globular CTD that binds to STAT1 and contains the C-NLS. GFP-fused P protein is functional in the inhibition of STAT1 (via the CTD) and nuclear export via the N-terminal NES, as well as in replication assays (which require the interaction of the N-terminal end of P protein with the polymerase (Lprotein)). GFP P protein has also been used to delineate P protein function, including in STAT1 binding and antagonism (e.g., [15,18,19,22,24]). Plasmids to express chimeric proteins variously encoding regions containing clusters of sequence diversity between the ABLV strains (Figure 1b; the N-terminal region (residues 52–143), Central region (residues 155–192), and C-terminal region (residues 242–284)) were generated using overlap PCR from the ABLVi-P and ABLVf-P plasmids. Plasmids to express GFP-fused negative control proteins from the RABV Challenge Virus Standard (CVS) strain (CVS-N protein (nucleoprotein) and CVS-PΔ30 protein (P protein deleted for the C-terminal 30 residues)), which lack antagonism/binding of STAT1, were described previously [22].

293T, Cos-7, and HeLa cells were maintained in Dulbecco’s Modified Eagle Medium (DMEM) with 10% fetal bovine serum (37 °C, 5% CO_2_). To express the P proteins, cells were transfected using Lipofectamine 2000 or 3000 (Thermo Fisher Scientific, Waltham, MA, USA), as previously described [22].

### 2.2. Reporter Gene Assays

Reporter gene assays were performed as previously described [16,22]. Briefly, constructs to express viral proteins of interest were cotransfected into 293T cells together with luciferase (luc) reporter plasmids for IFNα/STAT1/2-dependent signaling (pISRE-luc, in which firefly luc is under the control of the IFN-STAT1/2-sensitive IFN-stimulated response element (ISRE)) and transfection control (pRL-TK, from which *Renilla* luc is constitutively expressed). Cells were treated 6 h post-transfection without or with 1000 U/mL IFNα (PBL Assay Science, Piscataway, NJ, USA) for 16 h before measurement of luciferase activity, as previously described [22]. The ratio of firefly/*Renilla* luciferase activity was determined. Luciferase activity was then calculated relative to that for IFN-treated cells expressing CVS-N (negative control for antagonism of IFN signaling [22]).

### 2.3. Co-Immunoprecipitation (IP)

Cos-7 cells were transfected with plasmids to express viral proteins or controls for 24 h before coimmunoprecipitation (IP) using the GFP-trap system (Chromotek, Planegg-Martinsried, Germany) according to the manufacturer’s instructions. Where indicated, transfected cells were treated with 1000 U/mL of IFNα for 30 min or 16 h before IP. Complete protease inhibitor cocktail (Sigma, St Louis, MO, USA) and PhosSTOP (Sigma, St Louis, MO, USA) were included in lysis and wash buffers [14,16,22]. Proteins were detected by SDS-PAGE and immunoblotting (IB), using anti-pSTAT1 (Thermo Fisher Scientific, Waltham, MA, USA or Santa Cruz, Dallas, Texas, USA), anti-GFP (Sigma, St Louis, MO, USA or Abcam, Cambridge, UK), and anti-β-tubulin (Sigma, St Louis, MO, USA) antibodies.

### 2.4. Confocal Laser Scanning Microscopy (CLSM)

Cells were transfected for 24 h before treatment without or with 5.2 nM leptomycin-B (LMB, an inhibitor of exportin-1-mediated nuclear export; gift from Minoru Yoshida, RIKEN, Japan) for 3 h and imaging live in phenol-free DMEM with a heated chamber using a Nikon C1 inverted confocal laser scanning microscope. Images were analyzed using Fiji software (National Institutes of Health, NIH) to calculate the nuclear to cytoplasmic fluorescence ratio (Fn/c) corrected for background fluorescence for >40 cells/group, as described previously [14,29].

### 2.5. Statistical Analysis

Unpaired Student’s *t*-test was performed using GraphPad Prism 7.

## 3. Results and Discussion

### 3.1. Antagonism of IFN Signaling Differs between ABLVi and ABLVf P Proteins

ABLV circulates in frugivorous and insectivorous bats as two defined lineages [1,3,5,28]. Analysis using the Basic Local Alignment Search Tool (BLAST, NCBI) for ABLV from frugivorous bats (flying foxes) identifies 14 sequences for P protein, with the most divergent sequences showing >98% amino-acid identity. Analysis for ABLV from insectivorous bats identifies three sequences (two from bats and one from a horse infected by the bat variant) with the most divergent showing >99% identity; thus, there is minimal variation indicated within the population.

We generated plasmids to express ABLVi and ABLVf P proteins fused to GFP using isolates of ABLVi from yellow-bellied sheath tail bat and ABLVf from pteropid bat (GenBank Accession Numbers AF081020 and AF006497 for ABLVi and ABLVf, respectively). BLAST analysis of translated sequences of the cloned cDNA indicated a single residue difference for the cloned ABLVi P protein compared with the translated sequence from the AF081020 genome (E to K at position 84). There was also a single residue difference for the ABLVf P protein compared with the AF006497 sequence (L to P at position 263). Residues E84 and L263 in our cloned sequences were conserved in the other ABLVi or ABLVf sequences identified by BLAST, indicating that the differences may relate to errors in the original sequences.

**Figure 2:**
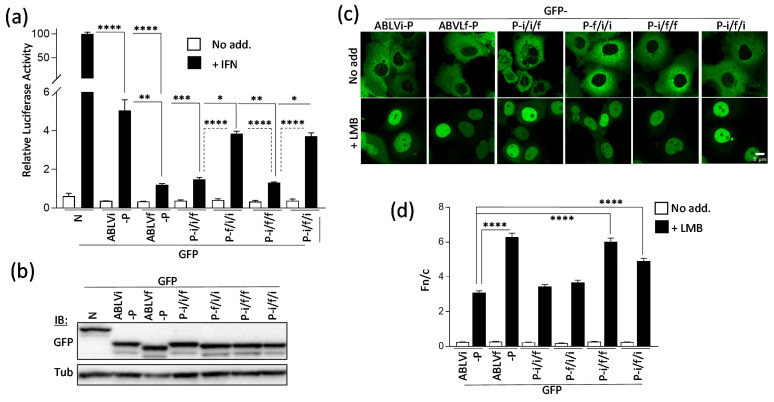
P proteins of ABLVi and ABLVf differ in STAT1 antagonist function and nuclear trafficking. (**a**) 293T cells were transfected to express the indicated proteins and with plasmids for the IFNα/STAT1-dependent dual-luciferase reporter assay before treatment with or without 1000 U/mL IFNα (16 h) and calculation of luciferase activity (ratio of firefly/*Renilla* activity; mean ± SD). **** *p* < 0.0001, *** *p* ≤ 0.001, ** *p* ≤ 0.01, * *p* ≤ 0.05 (Student’s *t*-test); data representative of ≥3 assays. (**b**) Expression of GFP and tubulin (Tub., loading control) in lysates from reporter assays was determined by immunoblotting (IB) using the indicated antibody. (**c**) Cos-7 cells expressing the indicated proteins were treated with or without 5.2 nM leptomycin-B (LMB) before analysis by confocal laser scanning microscopy (CLSM). (**d**) Images such as those in (**c**) were analyzed to calculate the ratio of nuclear to cytoplasmic fluorescence (Fn/c, mean ± SEM). **** *p* < 0.0001, *n* > 40 cells for each condition.

The ABLVi and ABLVf P proteins are 83.8% identical, with substitutions clustered broadly within three regions, corresponding to an N-terminal, a Central, and a C-terminal region (Figure 1a). To assess whether the changes impact IFN/STAT-antagonistic functions, we used a dual-luciferase reporter assay for IFNα-STAT1/2-dependent signaling [15,16,22]. 293-T cells were transfected to express the P proteins and with plasmids for the luciferase assay before treatment with IFNα and measurement of luciferase activity. Our previous analysis of P proteins of different lyssaviruses used ABLVf-P, which inhibited IFNα-STAT1/2 signaling to a similar extent as P proteins of several RABV strains and Mokola virus [22], and we confirmed that ABLVf-P significantly reduces IFN-activated luciferase expression compared to the control (Figure 2a). Consistent with the conservation of STAT1 antagonism between lyssavirus species [16,22], ABLVi-P also reduced luciferase activity [22] but to a significantly lesser extent than ABLVf-P, despite equivalent expression (Figure 2a,b). Comparable results were obtained using Cos-7 cells (Supplementary Figure S1). Thus, ABLVi-P is a less potent antagonist than ABLVf-P.

### 3.2. Nuclear Trafficking Differs between ABLVf and ABLVi P Protein

Antagonism of IFN/STAT1 signaling is associated with nucleocytoplasmic trafficking of P protein [8,9]. To determine whether differing antagonism correlates with altered trafficking, we analyzed cells transfected to express GFP-fused P proteins using confocal laser scanning microscopy (CLSM). In common with P proteins of other lyssaviruses and consistent with the conservation of the N-NES motif (Figure 1a; [22]), ABLVi and ABLVf P proteins were almost exclusively cytoplasmic at steady state (Figure 2c). Treatment of cells with leptomycin-B (LMB) to inhibit exportin-1 (which mediates N-NES-dependent nuclear export [20]) results in the nuclear accumulation of P protein in RABV-infected cells [9] and of transfected P protein of RABV and lyssaviruses [20,22], enabling the analysis of nuclear import activity. Consistent with the conservation of essential C-NLS residues (Figure 1a) [18,19], LMB treatment resulted in the nuclear accumulation of ABLVi-P and ABLVf P proteins (Figure 2c,d). However, the accumulation of ABLVf P protein, determined by calculating the nuclear to cytoplasmic fluorescence ratio (Fn/c) [14,22,25], significantly exceeded that of ABLVi P protein (Figure 2c,d). Comparable results were observed in HeLa cells (Figure S2). Thus, nuclear trafficking and IFN antagonism are maintained but differ in extent between ABLVi and ABLVf P proteins such that differing STAT1 antagonism might relate to nuclear localization activity.

### 3.3. Differing Nuclear Trafficking and IFN Antagonism Depend on Distinct Regions of ABLV P Protein

Key sequences mediating nuclear trafficking by full-length RABV P protein are the N-NES, C-NLS/C-NES, S210, and the DLC-AS (Figure 1A) [9,18,19,20,25]. With respect to STAT1 binding, residues W265 and M287 of the CTD were initially implicated, as they form part of a hydrophobic pocket (referred to as the “W-hole” [15,16,30]) in which naturally occurring substitutions between certain lyssaviruses (position 265, which is W in RABV P protein and G in DUVV P protein) or introduced mutations (W265G, M287V) inhibit STAT1 interaction [16]. These effects appear indirect, however, as the W-hole is distant to the STAT1 interaction surface recently identified in the CTD using nuclear magnetic resonance spectroscopy (NMR) [15]. All of these key residues/motifs are conserved between ABLVi and ABLVf (Figure 1a), consistent with retention of major functions and indicating that substitutions elsewhere impact as yet undefined regulatory sequences, or alter conformation.

To identify the regions responsible, we generated chimeric P proteins (Figure 1b; ABLVi/f/f, ABLVf/i/i, ABLVi/i/f, ABLVi/f/i) variously containing clusters of substitutions (the N-terminal, Central, and C-terminal regions) of ABLVi or ABLVf P (Figure 1). IFN/STAT1 reporter assays using these proteins (Figure 2a) indicated that the ABLVf-P C-terminal region is able to confer antagonist function comparable to that of wild-type ABLVf-P, indicating that changes in the CTD are the major determinant of the differing function in STAT1 inhibition. This is consistent with the CTD containing the STAT1 interface [15,17]. However, the data also indicated minor effects of changes in the ABLVi P protein N-terminal/Central regions, suggestive of an accessory impact of other regions.

In contrast, CLSM analysis indicated that the C-terminal region of ABLVf P protein is not sufficient to substantially enhance the nuclear import of ABLVi P protein (Figure 2 c,d). In fact, the ABLVf P protein Central region was the only region sufficient to substantially increase the nuclear import of ABLVi-P, with the N-terminal or C-terminal regions having little effect alone (Figure 2c,d). Thus, the Central region is a principal determinant of differing nuclear import (Figure 2c,d), although the recapitulation of nuclear import to levels comparable with wild-type ABLVf-P required both the Central and C-terminal regions of ABLVf P protein. These data indicate that differences in nuclear localization of ABLVf-P and ABLVi-P are not due to a discrete change in a specific interface with cellular trafficking machinery but result from substitutions in several regions, with changes to the Central region of primary importance. As the globular C-terminal domain contains the C-NLS, the data indicate that efficient activity of this sequence requires additional sequences in the Central region. Notably, the Central region lacks defined trafficking sequences for full-length P protein (Figure 1a), and key residues of the DLC-AS, C-NLS, or the regulatory PKC site (S210) are not altered between ABLVf and ABLVi P protein (Figure 1a), so the effect of substitutions is likely to involve modulation of previously unrecognized regulatory sequences or conformational effects impacting function/presentation of the NLS. Taken together, the distinct requirements for efficient IFN antagonism and nuclear trafficking, particularly with respect to the C-terminal region, indicate that differing antagonism does not result from altered nuclear import.

### 3.4. Differing Antagonistic Functions of ABLV P Protein Are Due to Altered Interaction with STAT1

**Figure 3:**
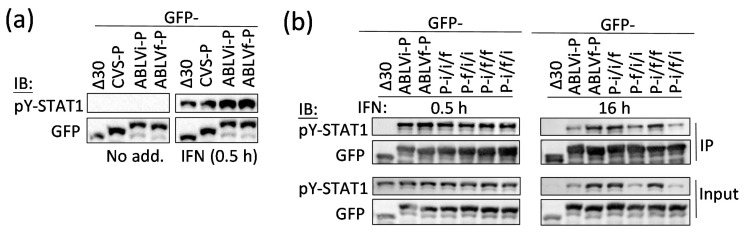
P protein–STAT1 interaction differs between ABLV P proteins and correlates with differing antagonist functions. (**a**) Cos-7 cells expressing the indicated proteins were treated with or without 1000 U/mL IFNα for 0.5 h before lysis and analysis by IB for Y701-phosphorylated STAT1 (pY-STAT1) and GFP. (**b**) Cells expressing the indicated proteins were treated for 0.5 and 16 h with IFN before IP for GFP and analysis of lysate (input) and IP by IB. Results representative of 3 experiments.

Our data indicated that differences in STAT1 antagonism by ABLV P proteins are dependent on the C-terminal region, which forms part of the CTD that binds to STAT1 [15,17]. We thus hypothesized that ABLVf and ABLVi P proteins might differ in interaction with STAT1. Efficient P protein–STAT1 interaction in cells requires IFN treatment, indicative of high-affinity binding to pY-STAT1 such that P protein selectively targets IFN-stimulated signaling processes [15,21,22]. We thus examined interactions of P proteins with pY-STAT1 by coimmunoprecipitation (IP) of GFP-fused P protein from cells following treatment with IFN for 0.5 h or 16 h (as previously described [22]) and analysis of lysates and IPs by immunoblotting (IB) (Figure 3).

pY-STAT1 was not detected in untreated cells but was clearly induced following 0.5 h treatment with IFN, with no inhibitory effect of P proteins of CVS RABV, ABLVi, or ABLVf compared with CVS-PΔ30 (a standard control lacking STAT1-binding activity [17,22]) (Figure 3a). This is consistent with previous reports that lyssavirus P proteins do not impair the phosphorylation of STAT1 at Y701 [22,31].

Following an early peak in Y701 phosphorylation after IFN treatment (c. 0.5–1 h), pY-STAT1 that has translocated to the nucleus to activate IFN-stimulated genes is inactivated by dephosphorylation by nuclear phosphatases [10,15,21,22,31]. This process is inhibited by P protein, likely due to the inhibition of DNA interaction/cytoplasmic retention of pY-STAT1 to antagonize signaling [10,15,21,22,31]. This results in the accumulation of pY-STAT1 in inhibitory complexes, presumably enabling sustained antagonism. We observed typical activation/dephosphorylation of STAT1 in cells expressing control protein, where pY-STAT1 accumulated at 0.5 h and was lost by 16 h IFN treatment (Figure 3b). At the 0.5 h time point, comparable levels of pY-STAT1 were detected in the lysates of cells expressing wild-type or chimeric P proteins (Figure 3b), and interaction detected by IP was also comparable. pY-STAT1 remained detectable in cells expressing all P proteins after 16 h IFN treatment, indicative of interaction (Figure 3b). However, levels of pY-STAT1 were clearly higher in cells expressing ABLVf-P or chimeras containing the ABLVf-P C-terminal region (P-i/i/f, P-i/f/f) compared with cells expressing ABLVi-P or chimeras containing the ABLVi-P C-terminal region (P-f/i/i, P-i/f/i), indicating reduced interaction by the latter. This was supported by analysis of IPs (Figure 3b). Thus, the differing IFN-antagonist functions of ABLVf and ABLVi P proteins appear to be due to altered interaction with STAT1 due to changes in the C-terminal region, proximal to the STAT-binding site in the CTD (Figure 1; [15,17]). However, as interaction with pY-STAT1 was observed for all proteins, the substitutions do not disable the STAT1-binding site (consistent with the conservation of critical STAT1 interaction residues (Figure 1a)) but rather impair interaction efficiency.

The finding that P proteins/chimeras bind well to pY-STAT1 after 0.5 h IFN treatment (at peak levels of pY-STAT1) with differences in proteins containing the ABLVi-P C-terminal region becoming apparent at 16 h indicates a reduced ability to retain pY-STAT1, probably relating to reduced affinity and an associated increase in dephosphorylation by cellular phosphatases. These observations are similar to recent findings using RABV P protein mutated at the W-hole, which strongly inhibits antagonism of STAT1 signaling. Mutated protein retained significant STAT1-binding/antagonistic activity at early time points after IFN treatment (albeit reduced compared with wild-type protein) and could not efficiently retain pY-STAT1 over extended incubation periods [15]. This is consistent with the W-hole not forming part of the STAT1-binding site, such that mutations reduce binding efficacy without preventing interaction. In contrast, mutation of the STAT1-binding site at key residues F209/D235 (Figure 1a) ablated pY-STAT1 binding at all time points [15]. Our data using ABLV P proteins support the idea that the antagonistic function of P protein depends not only on initial binding to pY-STAT1 but also on the retention of pY-STAT1 in inactive complexes by inhibiting cycles of phosphorylation/dephosphorylation.

The W-hole was initially implicated in P protein–STAT1 interaction because differences in this region between DUVV and RABV P protein correlated with altered STAT1 antagonism. However, residues of the recently defined STAT1-binding interface (I201-F209, D235-I237, L276-V277) are highly conserved between lyssaviruses, including DUVV (having only a single conservative substitution compared with RABV, D236 to E236) and ABLV P proteins (residues are absolutely conserved (see Figure 1a; [15])), consistent with critical roles. Thus, functional variation between species and lineages appears to involve changes at regulatory/modulatory sites.

Given the significance of STAT1 targeting in infection [15,16], it is unlikely that DUVV/ABLVi have adapted primarily to attenuate STAT1 antagonism, and both viruses are pathogenic [1,7]. Furthermore, STAT1 and importinα2 (which binds to the P protein C-NLS to mediate nuclear import [19]) show >96% identity and >98% sequence similarity, respectively, between *Pteropus alecto* (*P.alecto*) and human proteins. In addition, the JAK-STAT pathway is highly conserved, and analysis of STAT1 sequences indicated high homology between bat, human, mouse, pig, and monkey [32]. BLAST analysis of *P. alecto* STAT1 protein indicates >95% identity between STAT1 proteins of bats and >96% identity with human STAT1. Thus, it would appear unlikely that the changes arising between ABLVi and ABLVf P protein are due primarily to major differences in STAT1 structure/function between reservoir bat species. It is possible that other cellular/molecular differences, potentially involving distinct proteins associated with STAT1 signaling or differing post-translational modifications of STAT1 might be important. Alternatively, differences in the broader bat IFN system, such as divergent baseline levels of systemic IFN (a key characteristic in bat immunity [2]) and consequent activation of STATs, may also be important, altering the relative requirement for efficient STAT1 targeting in different hosts. However, given the multifunctional nature of P protein [8,9], it is possible that differences between bat hosts in requirements for other functions, such as rate of viral genome replication, might result in subtle changes to fine-tune the virus to the host. Thus, phenotypic changes in STAT1 antagonism by P protein may have emerged through the adaptation of other functions, with associated sequence/structural changes impacting STAT1 binding. Clearly, while our data on P proteins in human cells (a target of natural ABLV infection [1,5,6] with high STAT1 protein homology with bat species) indicate phenotypic divergence, we currently lack comparative data using cells of the reservoir hosts. The lack of an appropriate cell line for the natural reservoir that maintains ABLVi (yellow-bellied sheath tail bat) precluded such analysis here. This is particularly important with respect to STAT1 antagonism, as ABLVf P protein antagonistic activity toward IFN/STAT1 signaling is comparable to that of other lyssavirus P proteins [22], suggesting key divergence in ABLVi P protein. Future availability of such systems and, potentially, analysis in host animals should enable delineation of the molecular basis of the altered phenotype.

## 4. Conclusions

Taken together, our data indicate that the P proteins of the two established lineages of ABLV, which are associated with frugivorous and insectivorous bats, differ in immune evasion and nuclear trafficking functions. The effects on immune antagonism and nuclear import are independent, resulting from altered sequences in different regions of the proteins, indicative of distinct mechanisms including altered interactions with STATs (see summary in Table 1). These data suggest that adaptation to the different bat hosts includes changes in P protein, which has multiple roles in viral infection including in replication and immune evasion. These data are consistent with potential differences in the virus–host interface of different bat species.

## Figures and Tables

**Table 1:** Summary of the relative activity of ABLVf and ABLVi proteins and chimeras.

Function	P Protein					
	ABLVf	ABLVi	i/i/f	f/i/i	i/f/f	i/f/i
Nuclear Import	+++	+	+	+	+++	++
IFN antagonism	+++	+	+++	++	+++	++
STAT1-binding	+++	+	+++	+	+++	+

## Data Availability

Data are available in the article and Supplementary Materials.

## Supplementary Materials

The following are available online at https://www.mdpi.com/article/10.3390/v13050831/s1, Supplementary Figure S1. Cos7 cells were transfected to express the indicated proteins or empty vector (EV) and with plasmids for the IFNα/STAT1 reporter assay before treatment with or without IFNα and calculation of luciferase activity, as described in the legend of Figure 2. **** *p* < 0.0001. Supplementary Figure S2. HeLa cells expressing the indicated proteins were treated with or without LMB before analysis by CLSM and calculation of Fn/c (mean ± SEM), as described in the legend of Figure 2. **** *p* < 0.0001, n ≥ 40 cells for each condition.
