# Supplementary figures and images for: Phenotypic Divergence of P Proteins of Australian Bat Lyssavirus Lineages Circulating in Microbats and Flying Foxes
Source: Viruses. 2021 May 4;13(5):831. doi: 10.3390/v13050831 (PMC8147779; doi:10.3390/v13050831)

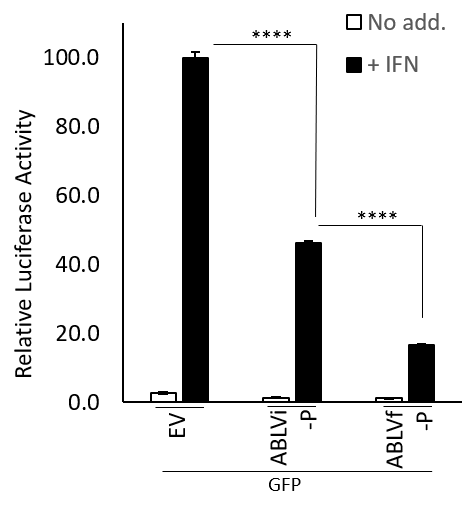

Supplement: Supplementary file 1 [file viruses-13-00831-s001.zip › Figure S1revised.png]

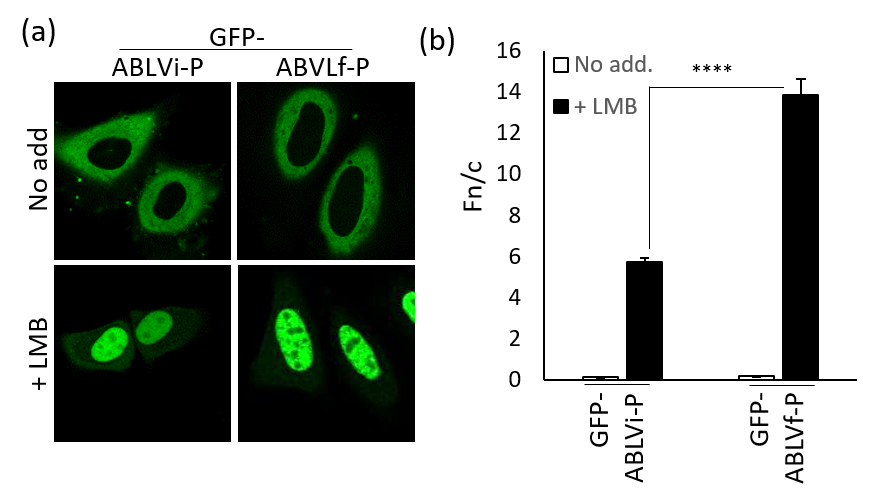

Supplement: Supplementary file 1 [file viruses-13-00831-s001.zip › Figure S2.png]
